# Supplementary material for: Triatomines outside the Americas: a comprehensive dataset for the global surveillance of Chagas disease vectors
Source: GigaByte. 2025 Aug 26;2025:gigabyte163. doi: 10.46471/gigabyte.163 (PMC12407106; doi:10.46471/gigabyte.163)
Supplement: Translation

# Translation: Triatominos por fuera de las Américas: conjunto de datos integral para la vigilancia global de los vectores de la enfermedad de Chagas

**Published:** 2025-08-20

**Authors:** Soledad Ceccarelli, Maria Eugenia Vicente, Qin Liu, Xiao-Nong Zhou, Di Wu, Agustin Balsalobre, Emiliano A. Bruno, S. Emilia Barboza, Romina Valente, Gerardo A. Marti

**Affiliations:** ^1^ https://ror.org/03qan4b12 Centro de Estudios Parasitológicos y de Vectores (CEPAVE-CCT-La Plata-CONICET-UNLP), B1900 La Plata, Buenos Aires, Argentina; ^2^ https://ror.org/03cqe8w59 Consejo Nacional de Investigaciones Científicas y Técnicas (CONICET), C1425FQB Buenos Aires, Argentina; ^3^ CIC, Comisión de Investigaciones Científicas de la Provincia de Buenos Aires, B1900 La Plata, Buenos Aires, Argentina; ^4^ https://ror.org/03wneb138 National Institute of Parasitic Diseases, https://ror.org/04wktzw65 Chinese Center for Disease Control and Prevention, Chinese Center for Tropical Diseases Research, WHO Collaborating Centre for Tropical Diseases, National Center for International Research on Tropical Diseases, Ministry of Science and Technology, Key Laboratory of Parasite and Vector Biology, Ministry of Health, Shanghai, 200025, China

## Abstract

La enfermedad de Chagas es causada por el *Trypanosoma cruzi*, que se transmite a mamíferos, incluyendo humanos, principalmente por insectos de la subfamilia Triatominae (Hemiptera: Reduviidae). Esta subfamilia incluye 159 especies en 18 géneros y cinco tribus. Aunque la mayoría de las especies se encuentran en el continente americano, aquí presentamos la primera compilación de ocurrencias de triatominos no americanos. Los datos (396 registros) corresponden a 16 especies de los géneros *Linschosteus* y *Triatom*a de África, Asia y Oceanía recolectadas entre 1926 y 2022, e incluyen registros verificados con coordenadas geográficas, fechas de recolección e información ecológica. Las principales novedades de nuestro conjunto de datos se refieren a: (i) actualizaciones temporales y geográficas de especies no americanas, (ii) registros de *T. rubrofasciata* cientos de kilómetros tierra adentro, y (iii) registros geográficos de las dos últimas especies de *Triatoma* descritas (*T. atrata* y *T. picta*). Nuestro recurso apoya la vigilancia global, el modelado ecológico y la evaluación de riesgos al proporcionar evidencia de vectores potenciales para el control de la enfermedad de Chagas fuera de las Américas.

## Descripción de los datos

### Contexto

La enfermedad de Chagas, causada por el protozoo *Trypanosoma cruzi* (Chagas, 1909) (NCBI:txid5693) (Kinetoplastida, Trypanosomatidae), se transmite principalmente a través de las heces de insectos vectores llamados triatominos (Hemiptera: Reduviidae: Triatominae), aunque también puede transmitirse de una persona gestante a su descendencia, por transfusiones de sangre o trasplantes de órganos infectados, y por transmisión oral mediante la ingesta de alimentos y/o bebidas contaminados. Estas múltiples rutas de transmisión hacen que la enfermedad de Chagas sea un problema importante de salud pública, principalmente en las Américas [1]. Sin embargo, los movimientos migratorios de personas infectadas con el parásito desde las América a otros continentes han contribuido a la expansión global del *Trypanosoma cruzi* [2], lo que resalta la necesidad de fortalecer la vigilancia entomológica en regiones previamente consideradas no endémicas [3].

Actualmente, la subfamilia Triatominae está formada por 156 especies actuales y tres fósiles, agrupadas en cinco tribus y 18 géneros [4, 5]. Luego de la última publicación integral sobre especies americanas de triatominos de Carcavallo y col. [6], se publica una base de datos actualizada e integrada sobre presencias de triatominos americanos [7]. Sin embargo, desde Ryckman y Archbold [8], no se ha realizado una integración ni actualización de la información sobre la distribución de especies de triatominos fuera de las Américas. En este contexto, el objetivo principal de este trabajo es describir las características de un conjunto de datos de presencias de triatominos no americanos (de ahora en adelante, el “conjunto de datos no americano”), destacando las actualizaciones e inclusiones más importantes. Este conjunto complementa la información actual sobre triatominos americanos (denominado “conjuntos de datos americanos”), que comprende dos bases con más de 35,000 registros y están disponibles en la plataforma Global Biodiversity Information Facility (GBIF) [9, 10] (Figura 1).

**Figura 1.** Distribución global de personas con Chagas y de especies de triatominos. Los polígonos verdes indican países con personas infectadas por el *Trypanosoma cruzi* según las últimas estimaciones oficiales, 2018 [11]. Los puntos naranjas representan registros de especies americanas de triatominos [9, 10] y los puntos rojos, representan registros de especies no americanas [12, 13].

 Este trabajo es el resultado de una revisión exhaustiva de información pública combinada con una colaboración interinstitucional sustancial. Este conjunto de datos puede contribuir, no solo a mejorar el conocimiento sobre la biodiversidad de las especies de triatominos fuera de las Américas, sino también en el diseño de estrategias mejoradas para la promoción de la salud y el control de vectores, para evaluar el estado actual y mostrar el impacto probable de la enfermedad de Chagas a escala global.

### Conjunto de datos de presencias de triatominos no americanos

En 1951, Lent asignó las especies del Indo-Pacífico solo a dos géneros, *Triatoma* Laporte, 1832 y *Linshcosteus* Distant, 1904 [14]. El presente estudio integra datos de 16 especies de ambos géneros de triatominos (Tabla  1), llegando a un total de 396 registros de presencias. En este conjunto de datos se han incluido registros de las dos últimas especies descritas de *Triatoma* (*T. atrata* Zhao & Cai sp. nov. 2023 y *T. picta* Zhao & Cai sp. nov., 2023 [5]). Estas incorporaciones, junto con las 126 especies incluidas en el conjunto de datos americano [9] y las 17 especies del conjunto de datos argentino [10], hacen un total de 158 especies de triatominos (*T. rubrofasciata* está incluida tanto en el nuevo conjunto de datos no americano como en el conjunto argentino debido a su distribución geográfica global). Exceptuando el caso particular de la especie *T. rosai* (Alevi, de Oliveira, Caris Garcia, Cesaretto Cristal, Grzyb Delgado, de Freitas Bittinelli, Visinho dos Reis, Ravazi, Bortolozo de Oliveira, Galvão, Vilela de Azeredo-Oliveira & Fernandez Madeira, 2020), que fue descrita en 2020 luego de la última actualización del conjunto de datos argentino (y por lo tanto no incluida), los registros de presencia de todas las especies americanas de triatominos descritas hasta la fecha [4, 5] están incluidos entre los tres conjuntos de datos (conjunto americano, argentino y no americano). 

**Tabla 1:** Clasificaciń taxonómica actual de las especies de triatominos no americanos (*T. rubrofasciata* también está presente en América) según la última clasificación taxonómica de Alevi *et al.* [4] y Zhao *et al.* [5].

Tribus	Género	Especie - nombre del/os descriptor/es - año	Número de registros en el conjunto de datos
Triatomini	*Linshcosteus*	*carnifex* Distant, 1904 *chota* Lent & Wygodzinsky, 1979 *confumus* Ghauri, 1976 *costalis* Ghauri, 1976 *kali* Lent & Wygodzinsky, 1979 *karupus* Galvao, Patterson, Rocha & Jurberg, 2002 *Linshcosteus sp.* Distant, 1904	1 1 8 9 4 2 1
	*Triatoma*	*amicitiae* Lent, 1951 *atrata* Zhao & Cai, 2023 *bouvieri* Larrousse, 1924 *cavernicola* Else & Cheong, 1977 *leopoldi* (Schouteden, 1933) *migrans* Breddin, 1903 *picta* Zhao & Cai, 2023 *pugasi* Lent, 1953 *rubrofasciata* (De Geer, 1773) *sinica* Hsiao, 1965 *Triatoma sp. Laporte, 1832*	1 3 4 2 6 12 20 1 317 3 1
		Número total de registros	**396**

Los géneros de triatominos incluidos en el conjunto de datos no americano están distribuidos en 34 países (o territorios de ultramar) de África (*n* = 14, incluyendo Reunión (Francia) y Azores (Portugal)), Asia (*n* = 15) y Oceanía (*n* = 5, incluyendo Hawái (Estados Unidos)), siendo Indonesia, China, India y Vietnam los países con la mayor cantidad de especies presentes (Tabla  2). Las seis especies del género *Linshcosteus* (*L. carnifex* Distant, 1904; *L. chota* Lent & Wygodzinsky, 1979; *L. confumus* Ghauri, 1976; *L. costalis* Ghauri, 1976; *L. kali* Lent & Wygodzinsky, 1979; y *L. karupus* Galvao, Patterson, Rocha & Jurberg, 2002) tienen registros (*n* = 26) solo en India (Tabla  2).

**Tabla 2:** Especies de triatominos presentes en cada país o territorio de ultramar.

Continent	Countries	Species
África	Angola Comoros Francia (Reunión) Madagascar Mali Mauricio República Centroafricana República Democrática del Congo Seychelles Sierra Leona Sudáfrica Sudán Tanzania Portugal (Azores)	*Triatoma rubrofasciata* *T. rubrofasciata* *T. rubrofasciata* *T. rubrofasciata* *T. rubrofasciata* *T. rubrofasciata* *T. rubrofasciata* *T. rubrofasciata* *T. rubrofasciata* *T. rubrofasciata* *T. rubrofasciata* *T. rubrofasciata* *T. rubrofasciata* *T. rubrofasciata*
Asia	Arabia Saudita Brunei Camboya China Filipinas Hong Kong India Indonesia Japón Malasia Myanmar Singapur Sri Lanka Tailandia Vietnam	*T. rubrofasciata* *T. migrans* *T. rubrofasciata* *T. atrata* *T. picta* *T. rubrofasciata* *T. sinica* *T. bouvieri* *T. migrans* *T. rubrofasciata* *T. rubrofasciata* *Linshcosteus carnifex* *L. chota* *L. confumus* *L. costalis* *L. kali* *L. karupus* *T. bouvieri* *T. migrans* *T. rubrofasciata* *T. leopoldi* *T. migrans* *T. pugasi* *T. rubrofasciata* *T. rubrofasciata* *T. cavernicola* *T. migrans* *T. rubrofasciata* *T. rubrofasciata* *T. rubrofasciata* *T. amicitiae* *T. rubrofasciata* *T. migrans* *T. rubrofasciata* *T. bouvieri* *T. migrans* *T. picta* *T. rubrofasciata*
Oceanía	Australia Estados Unidos (Hawai) Papúa Nueva Guinea República de kiribati Tonga	*T. leopoldi* *T. rubrofasciata* *T. leopoldi* *T. rubrofasciata* *T. rubrofasciata*

*Triatoma rubrofasciata* es la única especie tropicopolita de la subfamilia Triatominae (Figura 2). Se encontró con mayor frecuencia en ciudades portuarias, aunque el conjunto de datos aquí descrito presenta registros en algunos países (como India, China, Vietnam) donde esta especie ha sido encontrada entre 100 y 500 kilómetros tierra adentro.

**Figura 2.** Distribución geográfica global de *Triatoma rubrofasciata*. Los registros georreferenciados de África, Asia y Oceanía pertenecen al conjunto de datos no americano aquí descrito [12] y a datos mediados por GBIF [13]. Los registros de América pertenecen al conjunto de datos argentino [10].

 El rango temporal cubierto en el conjunto de datos va desde 1926 hasta 2022 (Figura 3). La información de fechas está disponible para el 73% de los registros (*n* = 289), y el 80% de estos registros (*n* = 239) corresponden a *T. rubrofasciata*. Esta especie fue la primera en ser descrita en el año 1773 [15], y el registro más antiguo incluido en este conjunto de datos corresponde al año 1963. Sin embargo, el 95,3% de los datos de esta especie se han recopilado en los últimos 10 años (Figura 4).

**Figura 3.** Distribución de frecuencia del número de registros por año de todas las especies de triatominos incluidas en el conjunto de datos no americano.

**Figura 4.** Distribución de frecuencia del número de registros por año de la especie *T. rubrofasciata*.

## Métodos

### Tipos de fuentes de información y recopilación de datos sobre especies de triatominos

Para construir el conjunto de datos, se obtuvo la información para cada especie de triatomino mediante una revisión detallada y exhaustiva. No se establecieron límites temporales específicos para así poder obtener la mayor cantidad posible de nuevos datos provenientes de tantos países como fuera posible.

En cuanto a la información publicada, se utilizaron varios repositorios bibliográficos en línea (como BioOne, Google Scholar, PLoS, PubMed, Scielo, ScienceDirect, Wiley) y se revisaron los mismos usando términos como “enfermedad de Chagas”, “Triatominae” junto con “África”, “Asia” y “Oceanía”, sin restricción idiomática.

Hubo una gran cantidad de datos correspondientes a China entre 2016 y 2018 proporcionados por colegas (coautores de este trabajo). La información geográfica forma parte de un artículo público [16], pero el conjunto de datos no estaba disponible.

### Proceso de georreferenciación de datos

Para asociar rigurosamente cada registro a una ubicación específica en el espacio geográfico, los datos deben contener información expresada en coordenadas geográficas (latitud y longitud). Si no estaban disponibles dichas coordenadas geográficas, se utilizó el nombre del sitio junto con información sobre divisiones administrativas para obtener una ubicación precisa mediante Google Earth [17]. Cuando solo estaban disponibles las coordenadas geográficas y el nombre del sitio (localidad), se completaron las divisiones administrativas correspondientes usando GeoLoc [18]. En el caso de los registros que solo tenían información a nivel de estado/provincia, no se añadieron las coordenadas geográficas. El datum (sistema de coordenadas y conjunto de puntos de referencia utilizados para localizar lugares en la Tierra) empleado para todos los registros geográficos fue el WGS84 (World Geodetic System 1984). El conjunto de datos final se construyó tras realizar un control de calidad de los datos.

### Descripción de los campos del conjunto de datos

Se recopiló toda la información relevante y disponible relacionada con cada especie de triatomino y se vincularon los datos a cada campo del conjunto de datos, incluyendo las características de los especímenes recolectados y de los sitios muestreados. Para describir mejor los campos (basados en los términos de Darwin Core [19]) utilizados para sistematizar la información, estos se agruparon en las siguientes seis categorías: (1) identificadores (incluyendo campos utilizados para identificar cada registro, por ejemplo, ID de presencia, código de la institución, idioma del recurso, referencias asociadas, etc.); (2) sistemático (incluyendo campos utilizados para la información sistemática, como nombre científico, autoría del nombre científico, rango taxonómico y observaciones sobre el taxón); (3) geográfico (incluyendo campos con información como divisiones administrativas, coordenadas geográficas, fuentes de georreferenciación, etc.); (4) temporal (incluyendo campos relacionados con la fecha del evento, como año, mes y día); (5) muestreo (incluyendo campos relacionados con el proceso de muestreo, como nombres del(os) colector(es), hábitat muestreado, protocolo y esfuerzo de muestreo, etc.); y (6) de individuos (campos relacionados con el número total, sexo y etapa de vida de los individuos muestreados). Las siguientes subsecciones proporcionan detalles sobre algunos de los campos mencionados que requieren aclaraciones específicas.

#### Campos sistemáticos

Cuando fue apropiado, en el campo “taxonRemarks” se incluyeron notas y/o referencias sobre sinónimos o transferencias formales de la especie descrita en el registro correspondiente.

#### Campos temporales

Cuando la información de un grupo de especímenes correspondía a un período de tiempo determinado pero con fechas específicas disponibles, los datos se dividieron en diferentes registros. Si no fue posible dividir los datos, cada registro incluía la información original del intervalo temporal (en años, meses o días) en el campo “eventDate” (por ejemplo, 2016/2018).

#### Campos de muestreo

El campo “hábitat” refiere al tipo de hábitat donde se recolectaron los triatominos, y se clasificó en tres categorías: domicilio, peridomicilio y silvestre. Cuando la información específica del hábitat estaba agrupada, el hábitat se expresa como una combinación de dos o tres de estas categorías (por ejemplo, domicilio–peridomicilio, domicilio–silvestre, peridomicilio–silvestre o domicilio–peridomicilio–silvestre). Para el campo “SamplingProtocols”, la información disponible se clasificó en dos categorías principales: (i) búsqueda activa, cuando la búsqueda involucraba personal especializado; y (ii) recolección pasiva, cuando se utilizaban diferentes tipos de trampas (por ejemplo, trampas de luz) y/o los datos provenían de proyectos de ciencia participativa (por ejemplo, iNaturalist).

## Validación de datos y control de calidad

El conjunto de datos fue sometido a un exhaustivo control de calidad. Primero, cada dato fue extraído por una persona y revisado por otras dos para garantizar su precisión y verificar que no hubiera registros duplicados. Posteriormente, los datos fueron revisados para evitar errores (por ejemplo, errores tipográficos, georreferenciación incorrecta, ubicaciones erróneas, sinónimos, errores en la ortografía de divisiones administrativas) que pudieran haber surgido durante la compilación o entrada de datos. Para corregir y eliminar errores tipográficos y errores ortográficos en los nombres de las divisiones administrativas, utilizamos el software OpenRefine (RRID:SCR_021305) [20], el cual ayuda a detectar estos tipos de errores en grandes conjuntos de datos. Todas las coordenadas geográficas fueron verificadas usando un software SIG libre (QGIS, RRID:SCR_018507 [21]) y Google Earth [17] con el fin de detectar errores de georreferenciación, como así también ubicaciones incorrectas, asegurando que cada punto correspondiera a una ubicación en el continente y país correcto. Cualquier coordenada atípica que estuviera geográficamente lejos de la distribución conocida de una especie dada fue estudiada en mayor profundidad para garantizar su precisión. Para detectar errores de sinónimos taxonómicos, utilizamos las revisiones más recientes sobre triatominos de especies actualmente válidas [4, 5]. Si algún nombre de especie parecía estar desactualizado, consultamos bibliografía actual o solicitamos la opinión de colegas.

Finalmente, mejoramos la calidad de nuestro conjunto de datos final utilizando el validador de datos de GBIF [22] con el fin de identificar y abordar posibles problemas antes de la publicación del conjunto de datos a través del Toolkit de Publicación Integrada (IPT, [23]).

## Potencial de reutilización

Dado que la información contenida en el conjunto de datos ha sido recopilada mediante diferentes procedimientos, esta compilación puede contener sesgos inherentes, los cuales deben ser considerados al utilizar los datos. La mayor parte de los datos (60%) proviene de artículos publicados en revistas científicas, junto con aquellos proporcionados por colegas (40%). Aunque los datos abarcan 34 países/territorios de ultramar, China tiene un volumen de datos mayor que los demás debido a que el número de registros está influenciado por la gran contribución de colegas de ese país, coautores de este trabajo (Qin Liu, Zhou Xiao-Nong y Di Wu). Al mismo tiempo, cabe mencionar tres notas importantes sobre los datos: (1) para la mayoría de las especies, su presencia pudo ser confirmada en países/estados donde los registros solo estaban disponibles en las décadas de 1960 y 1970; (2) en relación con lo anterior, los registros mediados por GBIF (es decir, iNaturalist), utilizados como complemento para mostrar la distribución global de *T. rubrofasciata* (Figura 2), demuestran la gran contribución que tienen los datos actuales provenientes de proyectos de ciencia participativa para la investigación científica y las políticas públicas; y (3) hacer públicos y disponibles los registros geográficos de las dos especies descritas más recientemente del género *Triatoma* (*T. atrata* Zhao & Cai sp. nov., 2023 y *T. picta* Zhao & Cai sp. nov., 2023 [5]).

Respecto al muestreo de los diferentes hábitats, reconocemos un posible sesgo a favor de los hábitats domiciliarios y peridomiciliarios, ya que estos son los hábitats de mayor importancia epidemiológica. Además, la escasez de datos sobre hábitats silvestres también resulta de la dificultad de los procedimientos de muestreo en la gran variedad de hábitats silvestres utilizados por los triatominos en lugares fuera de aquellas áreas donde comúnmente se reportan. Aquí es donde destacamos la utilidad en el uso de aplicaciones enmarcadas en proyectos de ciencia participativa para promover el aumento en los reportes de especies silvestres. Finalmente, cabe señalar que aproximadamente el 27% de los registros carecen de información temporal, y alrededor del 7% no tienen coordenadas geográficas; por lo tanto, recomendamos que cualquier análisis basado en este conjunto de datos utilice métodos que tengan en cuenta estos sesgos.

Por último, nos gustaría mencionar una referencia de un registro de *T. rubrofasciata* que consideramos que aunque posee información geográfica dudosa (Región de Murcia, España) [24], posiblemente como resultado de transporte pasivo, se debe considerar. Aunque el registro no se incluyó en el conjunto de datos, la evidencia disponible indica que éste es un modo plausible de transporte para estos insectos con mercancías. Adicionalmente, se demuestra la alta resistencia a la inanición que poseen estos insectos y el hecho de que la prevalencia más alta de enfermedad de Chagas en Europa ocurre en España. Por ello, consideramos que el establecimiento de una nueva población de este vector podría crear un nuevo escenario epidemiológico y debemos estar alertas.

A pesar de los sesgos de información descritos anteriormente, el conjunto de datos descrito en este artículo, junto con los conjuntos de datos complementarios de América, constituyen una valiosa recopilación de datos geográficos sobre triatominos americanos y no americanos, que es la más completa, actualizada e integrada hasta el momento. Por lo tanto, todos los conjuntos de datos mencionados aquí representan de la mejor manera el número de especies y países, y cuentan con coordenadas geográficas precisas. Dado que estos conjuntos de datos están alojados en un repositorio abierto y público, esperamos que contribuyan a cumplir con metas nacionales e internacionales, como promover el intercambio de información biológica, aumentar y mejorar la accesibilidad a dicha información, proporcionar datos biológicos producidos y compilados en varios países, y ampliar el conocimiento tanto sobre la biodiversidad como sobre los datos epidemiológicos relacionados con la enfermedad de Chagas.

## Data Availability

El conjunto de datos “Non-American triatomine occurrence data (Reduviidae:Triatominae)” ha sido publicado por el Centro de Estudios Parasitológicos y de Vectores (CEPAVE) [25] y está disponible en el repositorio GBIF bajo una licencia CC0 [12]. Datos adicionales están disponibles en GigaDB [26].

## Nota del editor

Este documento forma parte de una serie de artículos de publicación de datos en colaboración con GBIF y con el apoyo de TDR, el Programa Especial de Investigación y Capacitación sobre Enfermedades Tropicales de la Organización Mundial de la Salud, con el fin de publicar conjuntos de datos sobre vectores de enfermedades humanas [27].

## Declaraciones

### Aprobación ética

No aplica.

### Consentimiento para publicar

No aplica.

### Contribuciones de los autores

SC, MEV, AB y RV elaboraron el protocolo para la recolección de datos. SC, GM y MEV solicitaron datos no publicados a investigadores. MEV y SC realizaron el control de calidad general del conjunto de datos; QL, X-NZ y DW controlaron la calidad de los datos provenientes de China. SC redactó el primer borrador y todos los autores y autoras contribuyeron en el manuscrito. Todos los autores y autoras leyeron y aprobaron la versión final del manuscrito.

### Financiamiento

Esta investigación fue financiada por el subsidio “Fortalecimiento y promoción de proyectos de ciencia ciudadana” 2022, No 40 (Investigador principal GAM) del Fondo para la Investigación Científica y Tecnológica (FONCYT), Argentina.

## References

[1] World Health Organization (WHO). Integrating neglected tropical diseases into global health and development. In: Fourth WHO Report on Neglected Tropical Diseases. World Health Organization, 2017. https://apps.who.int/iris/handle/10665/255011.

[2] AvariaA, Ventura-GarciaL, SanmartinoM Population movements, borders, and chagas disease. Mem. Inst. Oswaldo Cruz, 2021; 116(1): e210151. doi:10.1590/0074-02760210151.PMC927318235830004

[3] GorlaDE, Xiao-NongZ, DiotaiutiL Different profiles and epidemiological scenarios: past, present and future. Mem. Inst. Oswaldo Cruz, 2022; 117: e200409. doi:10.1590/0074-02760200409.35613154 PMC9126320

[4] AleviKCC, de OliveiraJ, da Silva RochaD Trends in taxonomy of Chagas disease vectors (Hemiptera, Reduviidae, Triatominae): from Linnaean to integrative taxonomy. Pathogens, 2021; 10(12): 1627. doi:10.3390/pathogens10121627.34959582 PMC8706908

[5] ZhaoY, FanM, LiH Review of kissing bugs (Hemiptera: Reduviidae: Triatominae) from China with descriptions of two new species. Insects, 2023; 14: 450. doi:10.3390/insects14050450.37233078 PMC10231107

[6] CarcavalloRU, Curto de CasasSI, SherlockIA Geographical distribution and alti-latitudinal dispersion of Triatominae. In: CarcavalloRU, Galíndez GirónI, JurbergJ, LentH (eds), Atlas of Chagas’ Disease Vectors in the Americas. vol. 3, 1998; pp. 747–792.

[7] CeccarelliS, BalsalobreA, VicenteME American triatomine species occurrences: updates and novelties in the DataTri database. Gigabyte, 2022; 2022: 1–14. doi:10.46471/gigabyte.62.PMC993049936824504

[8] RyckmanRE, ArchboldEF. The triatominae and triatominae-borne trypanosomes of Asia, Africa, Australia and the East Indies. Bull. Soc. Vector Ecol., 1981; 6: 143–166.

[9] CeccarelliS, BalsalobreA, CanoME Datos de ocurrencia de triatominos americanos del Laboratorio de Triatominos del CEPAVE (CONICET-UNLP). Version 1.6. 2022; Centro de Estudios Parasitológicos y de Vectores (CEPAVE). Occurrence dataset. 10.15468/fbywtn. Accessed via GBIF.org on 4 June 2025.

[10] CeccarelliS, BalsalobreA, CanoME Datos de ocurrencia de triatominos presentes en Argentina del Laboratorio de Triatominos del CEPAVE (CONICET-UNLP). Version 1.2. 2020; Centro de Estudios Parasitológicos y de Vectores (CEPAVE). Occurrence dataset. 10.15468/ak8aax. Accessed via GBIF.org on 3 June 2025.

[11] OMS. Global distribution of cases of Chagas disease, based on official estimates. 2020; Available from: https://www.who.int/docs/default-source/ntds/chagas-disease/chagas-2018-cases.pdf?sfvrsn=f4e94b3b_2.

[12] CecarelliS, VicenteME, QinL Non-American triatomine occurrence data (Reduviidae: Triatominae). Version 1.0. 2025; Centro de Estudios Parasitológicos y de Vectores (CEPAVE). Occurrence dataset. 10.15468/qbfpxv.

[13] GBIF.org. GBIF Occurrence Download (5 June 2025). 10.15468/dl.cz4zmw.

[14] LentH. Triatominae das Regioes Oriental, Australiana, Etiopica e Paleartica, con descricao de uma nova especie (Hemiptera, Reduviidae). Rev. Bras. Biol., 1951; 11: 425–429.

[15] De GeerC. Memoires pour servir a l’histoire des insectes. Tome III. Stockholm: Pierrer Hesselberg, 1773; p. 696. doi:10.5962/bhl.title.14802.

[16] LiuQ, WuF, ShiY Distribution of Triatomines, the Vector of Chagas Disease-Southern China, 2016–2018. China CDC Wkly., 2020; 2(33): 629–633. doi:10.46234/ccdcw2020.174.34594725 PMC8392960

[17] Google: Google Earth. https://www.google.com.ar/intl/es/earth/. Accessed 3 June 2025.

[18] SpeciesLink: GeoLoc. https://splink.cria.org.br/geoloc?criaLANG=pt. Accessed 4 June 2025.

[19] WieczorekJ, BloomD, GuralnickR Darwin Core: an evolving community-developed biodiversity data standard. PLoS One, 2012; 7(1): e29715. doi:10.1371/journal.pone.0029715.22238640 PMC3253084

[20] OpenRefine. https://openrefine.org/. Accessed 4 June 2025.

[21] QGIS Development Team. QGIS Geographic Information System. Open Source Geospatial Foundation Project. https://qgis.org/.

[22] Global Biodiversity Information Facility (GBIF). Data Validator. https://www.gbif.org/tools/data-validator. Accessed 5 June 2025.

[23] Global Biodiversity Information Facility (GBIF). IPT: The Integrated Publishing Toolkit. https://www.gbif.org/en/ipt. Accessed 5 June 2025.

[24] CollantesF, Campos-SerranoJF, Ruiz-ArrondoI. Accidental importation of the vector of Chagas disease, *Triatoma rubrofasciata* (De Geer, 1773) (Hemiptera, Reduviidae, Triatominae), in Europe. J. Vector Ecol., 2023; 48(1): 63–65. doi:10.52707/1081-1710-48.1.63.37255361

[25] CecarelliS, VicenteME, QinL Non-American triatomine occurrence data (Reduviidae: Triatominae). Version 1.1, 2025. Centro de Estudios Parasitológicos y de Vectores (CEPAVE). Occurrence dataset. 10.15468/qbfpxv. Accessed via GBIF.org on 8 August 2025.

[26] CeccarelliS, VicenteME, QinL Supporting data for “Triatomines outside the Americas: a comprehensive dataset to the global surveillance of chagas disease vectors”. GigaScience Database, 2025; 10.5524/102748.PMC1240710640909334

[27] Vectors of Human Disease Series. *GigaByte*. 2022; 10.46471/GIGABYTE_SERIES_0002.
